# Pregnancy and Inflammatory Rheumatological Diseases: A Single-Center Retrospective Cohort Study

**DOI:** 10.7759/cureus.47277

**Published:** 2023-10-18

**Authors:** Abeer A Alkhodier, Abdurhman S Alsaif, Norah H Alqntash, Rakan B Alanazi, Ghaida Alotaibi, Abdulrahman Alrashid

**Affiliations:** 1 College of Medicine, King Khalid University Hospital, King Saud University, Riyadh, SAU; 2 College of Medicine, King Saud Bin Abdulaziz University for Health Sciences, Riyadh, SAU; 3 Clinical Sciences, Princess Nourah bint Abdulrahman University College of Medicine, Riyadh, SAU; 4 Pathology, King Khalid University Hospital, King Saud University, Riyadh, SAU; 5 Rheumatology, King Abdulaziz Medical City, Riyadh, SAU

**Keywords:** sjogren syndrome, rheumatiod arthritis, congenital heart block, pregnancy outcome, systemic lupus erythematosus

## Abstract

Background

Rheumatic diseases pose risks to pregnant women, leading to complications like preterm birth, congenital heart block, and pregnancy loss. These diseases are expected to deteriorate during pregnancy and further in the postpartum period. The impact of these diseases on the pregnancy will add further burden on the patient, fetus, physician, and healthcare system. Advances in diagnosis and treatment have improved outcomes making them similar to that of healthy women, but close follow-up in a multidisciplinary clinic is essential. The objective of this study is to study the outcome of pregnancy in women with rheumatological disease and the behavior of the disease during pregnancy.

Methods

A retrospective cohort study was conducted in King Abdulaziz Medical City (KAMC) in Riyadh, Saudi Arabia, to compare the outcomes of pregnancy across three rheumatological diseases: Sjogren syndrome (SS), systemic lupus erythematosus (SLE), and rheumatoid arthritis (RA) from 2016 to 2021. A total of 128 pregnancies in 107 women with rheumatological diseases were included in this study. Pregnancy measures and outcomes were investigated by assessing maternal health, fetal health, and pregnancy complications, specifically maternal disease activity, medications to control the disease, infection, preterm birth, birth weight, abortions/stillbirths, mode of delivery, bleeding, preeclampsia, congenital heart block, and neonatal lupus.

Results

There were 55 patients with RA (63 RA pregnancies), 44 with SLE (54 SLE pregnancies), and eight with primary SS (11 SS pregnancies). In most of the pregnancies (n= 108; 95.58%), the patients were in clinical remission before pregnancy. Lupus nephritis, which was in remission before pregnancy, has been reported in nine (16.67%) out of 54 SLE pregnancies. Vaginal delivery was the most common mode of delivery (n=87; 67.97%). On the other hand, there were 38 cesarean sections (29.69%). Rheumatological disease flares occurred in 10 pregnancies (7.87%). One hundred and twenty-two live births were delivered. Preterm infants were born in 25 pregnancies (20.16%), and 16 (13.22%) of the newborns needed neonatal intensive care unit (NICU) care. Interestingly, congenital heart block (CHB) was found in five (12.2%) neonates out of 41 anti-SS-related antigen A (anti-SSA) positive mothers; one of those five died from heart block. Eleven neonates were delivered with positive serology, and five were diagnosed with neonatal lupus.

Conclusion

The outcome of pregnancy in patients with rheumatological disease is favorable. A multidisciplinary team approach and close clinical follow-up are the cornerstone for such success. A small dose of prednisolone (5 mg or less) is safe and will not have a negative impact on maternal or fetal health. CHB is a concern for pregnant women with positive anti-SSA.

## Introduction

Rheumatic diseases are a broad spectrum of disorders that involve many tissues. They can be classified according to their nature as autoimmune, inflammatory, or degenerative/metabolic disorders. Autoimmune rheumatic diseases (ARDs) are a diverse group of conditions that affect predominantly, but not only, the muscles and joints. These diseases are more common in females. ARDs include systemic lupus erythematosus (SLE), rheumatoid arthritis (RA), Sjogren syndrome (SS), and many others. They are considered challenging during the early stages as they present with nonspecific symptoms [1].

Historically, pregnancy in women with rheumatic diseases such as RA, SLE, and SS has presented a challenge to physicians due to the disease’s activity and the effect of pregnancy on the disease. The clinical spectrum of these diseases varies from one to another. For example, SLE can cause various manifestations such as mucocutaneous, hematologic, renal, and neurologic manifestations [2,3]. Moreover, these rheumatic diseases can lead to serious complications, particularly in pregnant women. For example, preterm birth, congenital heart block, which is the manifestation of neonatal lupus, and pregnancy loss are the most common pregnancy complications. Most of these diseases are expected to deteriorate during pregnancy and further in the postpartum period [3-5]. According to recent studies, there was a 25-65% increase in the rate of lupus flares associated with pregnancy [6]. However, this is not the case in women with RA, as 75% of them experience remission during pregnancy. Unfortunately, 80% of the patients will relapse within three months after delivery [5]. In addition to the previously mentioned complications, they can be associated with secondary syndromes, such as antiphospholipid syndrome (APS) [7]. In general, pregnancy complications and their severity vary according to the disease stage, the presence of organ damage and its severity, and treatment [3-7]. The impact of these diseases on the pregnancy will add further burden on the patient, fetus, physician, and healthcare system.

Generally, it is considered safe to use hydroxychloroquine (HCQ) for the treatment of autoimmune rheumatic disorders during pregnancy, and the continuation of this drug during pregnancy is commonly advised for the purpose of improving both pregnancy outcomes and disease management [8]. Moreover, it was found in another study that the use of corticosteroids, azathioprine, and HCQ is safe during pregnancy, and no adverse effects on fetuses were reported despite many years of use [9]. Additionally, it has been demonstrated that a low dose of aspirin alone or combined with enoxaparin improved outcomes in many cases of SLE and antiphospholipid syndromes [10].

Furthermore, women with rheumatic diseases may not necessarily experience high-risk pregnancies. Counseling prior to conception provides the ideal circumstances; since all past obstetric history, disease activity, presence of organ damage, serology profile, and further medical history can be evaluated, allowing personalized discussion of the possible pregnancy complications. Additionally, any medication adjustments that may be necessary can be made. In these women, close surveillance during pregnancy and postpartum, as well as a tailored management approach, are key to obtaining a successful pregnancy [11].

Overall, pregnancies for patients with SS, SLE, and RA have a high risk of both maternal and fetal morbidity and mortality. According to the best of our knowledge, this is the first study at the national level focusing on the pregnancy outcome in patients with rheumatological diseases; thus, it will serve as a quality indicator for the current clinical care for these patients, discover challenges, and highlight areas of potential improvements.

## Materials and methods

This was a retrospective cohort study at King Abdulaziz Medical City (KAMC) in Riyadh, Saudi Arabia. We compared the outcomes of pregnancy across three common rheumatological diseases, SS, SLE, and RA, from 2016 to 2021. The study was approved by the Institutional Review Board of King Abdullah International Medical Research Center, Ministry of National Guard-Health Affairs, Riyadh, Kingdom of Saudi Arabia (approval number: #RSS21R/013/07).

Study subjects meeting the following inclusion criteria were enrolled: all pregnant women with RA, SLE, and SS from 2016 to 2021, rheumatology patients referred to the obstetric clinics from 2016 to 2021, and all pregnant women who were seen in the obstetric clinic at KAMC with confirmed diagnoses of rheumatological diseases by a rheumatologist. The study exclusion criteria were any patients with non-inflammatory rheumatological diseases, patients with inflammatory rheumatological diseases other than RA, SLE, and SS, non-pregnant patients with rheumatological diseases, and patients whose rheumatological disease was not confirmed by a rheumatologist.

Pregnancy measures and outcomes were investigated by assessing maternal health, fetal health, and pregnancy complications, specifically, maternal disease activity, medications to control the disease, infection, preterm birth, birth weight, abortions/stillbirths, mode of delivery, bleeding, preeclampsia, congenital heart block, and neonatal lupus. Regarding disease activity, a flare is defined as per a clinician’s assessment either clinically (presence of active disease manifestation clinically) or biochemically (high anti-DNA, low C3 and C4, and elevated inflammatory markers) in correlation with clinical assessment.

All variables were collected through an examination of patient electronic records in the BESTCare system. The behavior and the outcome of both the pregnancy course and the rheumatological disease were closely observed. Also, the obstetrics database was used. The data were collected by reviewing the patient's electronic medical records (EMR) using the BESTCare system in National Guard Health Affairs to extract the dependent variable, which is the outcome of common rheumatological diseases along with its associated independent variables like maternal demographic characteristics, history of rheumatological diseases, treatment, obstetric history, smoking habits, comorbidities, and presence of antiphospholipid antibodies. All patients who met our inclusion criteria were included. Then the data was stored in Excel sheets (Microsoft Corporation, Redmond, Washington, United States) by five of the six investigators, and then confirmed and revised by the sixth investigator. To ensure the integrity of the data, backup pressure was followed. Microsoft Excel was used for data entry whereas data analysis was done using the latest John's Macintosh Project (JMP) data analysis software (JMP Statistical Discovery LLC, Cary, North Carolina, United States). Descriptive statistics were applied to the data. Mean and standard deviation were used for continuous data. Frequency and percentage were used to analyze and present the categorical data. Analytic statistics were applied. A chi-square test was performed on categorical data. A p-value of less than 0.05 was considered significant.

## Results

Maternal clinical backgrounds

A total of 128 pregnancies in 107 women with rheumatological diseases were included in this retrospective cohort study. Several patients experienced two or more pregnancies during the study period. The median age of the patients was 34 years and the minimum and maximum were 20 and 45, respectively. One hundred and twenty-four pregnancies (96.88%) were spontaneous, while four (3.13%) were in vitro fertilization (IVF). Nineteen patients had more than one pregnancy during the study period. There were 55 patients with RA (a total of 63 pregnancies), 44 with SLE (a total of 54 pregnancies), and eight with primary SS (a total of 11 pregnancies). Notably, out of the total pregnancies, one pregnancy was with overlapped RA and SLE (Rhupus syndrome, a rare combination of RA and SLE). In 61 pregnancies (47.66%), the patients had no comorbidities, and pregnancies with patients having one, two, or three comorbidities were 38 (29.69%), 18 (14.06%), and 11 (8.59%), respectively. Hypothyroidism was found in 15 cases (11.72%), with SLE having a particularly higher prevalence (10 out of 15). Diabetes was found in 10 (7.81%), hypertension in eight (6.25%), and other comorbidities in 48 (37.5%) pregnancies. Lupus nephritis, which was in remission before pregnancy, was found in nine (16.67%) out of 54 SLE pregnancies from 44 SLE patients. Medications used during pregnancies were restricted to prednisolone (77 patients) with a dose of 2.5-20 mg daily and a mean of 5.23 mg daily, HCQ (89 patients) with a dose of 200-400 mg daily and a mean of 260.67 mg daily, enoxaparin (25 patients) with a dose of 40-80 mg daily and a mean of 50 mg daily, and azathioprine (24 patients) with a dose of 50-225 mg daily and a mean of 109.5 mg daily for the treatment of rheumatological diseases (Table 1).

**Table 1:** Patients’ general characteristics and treatments SLE: systemic lupus erythematosus; RA: rheumatoid arthritis; SS: Sjogren syndrome; IVF: in vitro fertilization; HCQ: hydroxychloroquine (NA)*: Not Available, these frequencies were missing

	Total (n= 128)	SLE (n= 54)	RA (n= 63)	SS (n= 11)
General characteristics				
Maternal age, median (min-max) (years old)	34 (20-45)	30.5 (20-41)	35 (24-44)	37 (24-45)
Diabetes, no. (%)	10 (7.81)	6 (11.11)	3 (4.76)	1 (9.09)
Hypothyroidism, no. (%)	15 (11.72)	10 (18.52)	3 (4.76)	2 (18.18)
Hypertension, no. (%)	8 (6.25)	5 (9.26)	3 (4.76)	0 (0)
Lupus nephritis, no. (%)	-	9 (16.67)	0 (0)	0 (0)
Smoking, no. (%)	0 (0)	0 (0)	0 (0)	0 (0)
IVF, no. (%)	4 (3.13)	1 (1.85)	1 (1.59)	2 (18.18)
Treatment				
Prednisolone, no. (%) (NA)*	77 (62.6) (5)*	41 (78.85) (2)*	35 (55.56) (0)*	1 (12.5) (3)*
Dosage of Prednisolone, mean (min-max) (mg/day)	5.23 (2.5- 20)	5.12 (2.5-7.5)	5.36 (2.5-20)	5 (5-5)
HCQ, no. (%) (NA)*	89 (72.36) (5)*	43 (82.69) (2)*	41 (65.08) (0)*	5 (62.5) (3)*
Dosage of HCQ, mean (min-max) (mg/day)	260.67 (200-400)	283.72 (200-400)	236.59 (200-400)	260 (200-400)
Enoxaparin, no. (%) (NA)*	25 (20.33) (5)*	18 (34.62) (2)*	5 (7.94) (0)*	2 (25) (3)*
Dosage of Enoxaparin, mean (min-max) (mg/day)	50 (40-80)	48.33 (40-80)	48 (40-60)	70 (70-70)
Azathioprine, no. (%) (NA)*	24 (20) (8)*	21 (40.38) (2)*	3 (5) (3)*	0 (0) (3)*
Dosage of Azathioprine, mean (min-max) (mg/day)	109.5 (50-225)	114.43 (50-225)	75 (50-100)	0 (0)

Pregnancy, maternal, and fetal outcomes

During the 128 pregnancies, 24 complications (e.g., thrombosis, preeclampsia, and others) were found. Thrombosis was reported in 1 SS pregnancy (0.78%). Preeclampsia was reported in 1 SS and 3 SLE pregnancies (3.13%), and 19 other pregnancy complications were reported (Table 2).

**Table 2:** Pregnancy complications SLE: systemic lupus erythematosus; RA: rheumatoid arthritis; SS: Sjogren syndrome; IUGR: intrauterine growth restriction; GDM: gestational diabetes mellitus

	Total (n= 128)	SLE (n= 54)	RA (n= 63)	SS (n= 11)
Thrombosis, no. (%)	1 (0.78)	0 (0)	0 (0)	1 (9.09)
Preeclampsia, no. (%)	4 (3.13)	3 (5.56)	0 (0)	1 (9.09)
Others				
Anemia, no. (%)	3 (2.34)	2 (3.7)	1 (1.59)	0 (0)
Oligohydramnios, no. (%)	1 (0.78)	1 (1.85)	0 (0)	0 (0)
Itchy skin lesion, no. (%)	1 (0.78)	1 (1.85)	0 (0)	0 (0)
Raised liver enzymes, no. (%)	1 (0.78)	1 (1.85)	0 (0)	0 (0)
Intrahepatic cholestasis, no. (%)	1 (0.78)	1 (1.85)	0 (0)	0 (0)
Bleeding, no. (%)	5 (3.91)	2 (3.7)	1 (1.59)	2 (18.18)
IUGR, no. (%)	6 (4.69)	1 (1.85)	5 (7.94)	0 (0)
GDM, no. (%)	1 (0.78)	0 (0)	1 (1.59)	0 (0)
Total, no. (%)	24 (18.75)	12 (22.22)	8 (12.70)	4 (36.36)

Out of the 122 live births delivered, 52 were male and 70 were female newborns. The median gestational age was 38 weeks for all live births, and the median birth weight was 2.70 kg. Controlling the three diseases in 71 pregnancies with a small dose of prednisolone (5 mg or less) during pregnancy was safe and did not show any significant impact on neonatal birth weight. In the remaining 6 pregnancies, prednisolone’s dose was more than 5 mg and thus not considered small. Regarding the subgroup analysis, the median birth weight of SLE, RA, and SS was 2.8 kg, 2.75 kg, and 2.7 kg, respectively. Forty-three infants (34.68%) were low birth weight (less than 2.5 kg) and three infants (2.42%) were very low birth weight (less than 1.5 kg) (Table 3).

**Table 3:** Pregnancy outcome, maternal and fetal adverse events SLE: systemic lupus erythematosus; RA: rheumatoid arthritis; SS: Sjogren syndrome; C-section: cesarean section; PROM: premature rupture of membranes; NICU: neonatal intensive care unit (NA)*: Not Available, these frequencies were missing

	Total (n=128)	SLE (n=54)	RA (n=63)	SS (n=11)
Pregnancy outcome				
Live births, no. (%)	122 (95.31)	52 (96.3)	60 (95.24)	10 (90.91)
Gestational weeks, median (min-max) (weeks)	38 (27-41)	37 (30-41)	38 (27-41)	37 (34-40)
Birth weight, median (min-max) (kg)	2.7 (1-4)	2.8 (1.09-3.8)	2.75 (1-4)	2.7 (1.5-3.28)
Maternal adverse events				
Abortion/stillbirths, no. (%) (NA)*	6 (4.69) (0)*	2 (3.7) (0)*	3 (4.76) (0)*	1 (9.09) (0)*
Disease flare, no. (%) (NA)*	10 (7.87) (1)*	1 (1.85) (0)*	8 (12.9) (1)*	1 (9.09) (0)*
C-section, no. (%) (NA)*	38 (29.69) (0)*	20 (37.04) (0)*	12 (19.05) (0)*	6 (54.55) (0)*
PROM, no. (%) (NA)*	4 (3.13) (0)*	1 (1.85) (0)*	2 (3.17) (0)*	1 (9.09) (0)*
Postpartum eclampsia, no. (%) (NA)*	0 (0) (0)*	0 (0) (0)*	0 (0) (0)*	0 (0) (0)*
Postpartum preeclampsia, no. (%) (NA)*	3 (2.34) (0)*	2 (3.7) (0)*	0 (0) (0)*	1 (9.09) (0)*
Postpartum bleeding, no. (%) (NA)*	3 (2.34) (0)*	1 (1.85) (0)*	1 (1.59) (0)*	1 (9.09) (0)*
Postpartum infection, no. (%) (NA)*	3 (2.34) (0)*	2 (3.7) (0)*	0 (0) (0)*	1 (9.09) (0)*
Fetal adverse events				
Low birth weight infants, no. (%) (NA)*	43 (34.68) (4)*	19 (35.85) (1)*	20 (32.79) (2)*	4 (40) (1)*
Very low birth weight infants, no. (%) (NA)*	3 (2.42) (4)*	2 (3.77) (1)*	1 (1.64) (2)*	0 (0) (1)*
Preterm infants, no. (%) (NA)*	25 (20.16) (4)*	14 (26.42) (1)*	8 (13.11) (2)*	3 (30) (1)*
Care in NICU, no. (%) (NA)*	16 (13.22) (7)*	7 (13.73) (3)*	5 (8.2) (2)*	4 (44.44) (2)*
Neonate with (+) serology, no. (%) (NA)*	11 (9.32) (10)*	10 (20) (4)*	1 (1.67) (3)*	0 (0) (3)*
Neonatal lupus, no. (%) (NA)*	5 (4.17) (8)*	5 (9.8) (3)*	0 (0) (3)*	0 (0) (2)*
Congenital heart block, no. (%) (NA)*	5 (4.5) (16)*	3 (5.77) (2)*	0 (0) (12)*	2 (22.22) (2)*

In 63 pregnancies, a previous abortion was reported, and in the majority (n=42; 66.67%), it happened once. Recurrent abortions were as follows: two previous abortions were reported in 17 pregnancies (26.98%), three previous abortions in three pregnancies (4.76%), and four previous abortions in one pregnancy (1.59%). In the period between 2016 and 2021, there were only six abortions/stillbirths (three abortions and three stillbirths). Delivered babies were 125 (97.65%); live births were 122 (95.31%) and stillbirths were three (2.34%). Vaginal delivery was 87 (67.97%); it was the most common mode of delivery with its two forms: 78 spontaneous (60.94%) and nine induced (7.03%).

On the other hand, cesarean sections were 38 (29.69%). In other words, almost one-third of the pregnancies ended with cesarean sections with the following percentages: SLE (37.04%), RA (19.05%), and SS (54.55%). Rheumatological disease flares occurred in 10 (7.87%). The manifestations included eight RA cases as joint pain; two had PROM as well, one ended with premature delivery at 33 weeks, and one ended with a terminated pregnancy at 26 weeks. One SLE flared up as joint pain and low complement levels (C3 and C4), and one SS as joint pain (Table 3). In most of pregnancies (n=108; 95.58%), the patients were in clinical remission before pregnancy. With regards to the few flare-ups in RA patients, they were reported during pregnancy and/or postpartum; nonetheless, they were not statistically significant (p-value=0.22). 

Complications experienced at the end of the pregnancies and following delivery (e.g., premature rupture of membranes (PROM), postpartum infection, postpartum bleeding, and postpartum preeclampsia) were noted in 13 pregnancies (10.15%). Moreover, preterm infants were born in 25 pregnancies (20.16%) (14 SLE, eight RA, and three SS), and 16 infants (13.22%) required neonatal intensive care unit (NICU) care. Eleven neonates were delivered with positive serology, and five of them were diagnosed with neonatal lupus by a pediatric rheumatologist (Table 3).

Interestingly, congenital heart block (CHB) was found in five neonates (12.2%) out of 41 anti-SS-related antigen A (anti-SSA) positive mothers; one out of those five died from heart block (Table 4). No cases of CHB were reported in anti-SSA-negative patients. 

**Table 4:** Congenital heart block in positive anti-SSA mothers Anti-SSA: anti–Sjögren's-syndrome-related antigen A

	SSA Positive	Percentage (%)
Heart block	5	12.20
No heart block	36	87.80
Total	41	100

## Discussion

By investigating 128 pregnancies in 107 women with rheumatological diseases in a retrospective cohort study, we found that five (12.20%) out of 41 neonates from anti-SSA-positive pregnancies (three with SLE mothers and two with SS mothers) had CHB. Anti-SSA-positive women are more likely to contract a CHB than other women having rheumatological diseases without positive anti-SSA.The risk association between anti-SSA and CHB has been well established in prior research, and our results could support this risk, aligning with multiple studies [12-14]. Although the percentage is quite higher than the international figure, this does not reflect the exact incidence because this is a single-center experience with a small sample size. In addition, KAMC is a tertiary-level center and tends to accept complicated medical cases rather than simple pregnancies. Furthermore, this study revealed that 22.22% of neonates born to mothers with SS developed CHB, as did 5.77% of neonates born to mothers with SLE and 0% of neonates born to mothers with RA. Our data may indicate that SS increases the risk of developing CHB more than SLE and RA. This finding is consistent with a previous study that reported that women with anti-Ro/SSA-positive pSS or other autoimmune diseases have a higher risk of CHB compared to those with SLE. Although the association between SS and the increased risk of CHB has been widely accepted, the exact mechanism of this association remains to be defined [12].

Furthermore, patients with rheumatic disease are at a higher risk of developing complications during pregnancy than the healthy population. These pregnancy complications include intrauterine growth restriction (IUGR), preeclampsia, anemia, venous thromboembolisms, disease flare, and others [15]. It has been reported in a previous study that the likelihood of flare-ups during pregnancy is lower when the condition is in remission at least six months before conception [16]. Interestingly, most women in this study were in remission at conception, and disease flare-ups were insignificant. The results of this study were in line with those of the previous study. In the current study, 10 pregnancies (7.87%) were found to have flares with eight being RA pregnancies, one being an SS pregnancy, and one being an SLE pregnancy. RA pregnancies with a disease flare accounted for 12.9% of all RA pregnancies, which is lower than that reported by van den Brandt et al., in which 61 (29%) pregnant RA patients had disease flares [17]. One SS pregnancy with disease flare was reported in the present study with a percentage of 9.09%, which was relatively close to another study that reported four flares (10%) during peripartum [18]. As for SLE flares, the current study had one pregnancy with a flare (1.85%), which is lower than that presented by Carvalheiras et al., in which 16 pregnancy-associated flares were reported (31%) [19]. IUGR was more prevalent in patients with RA in this study. Six IUGR cases were reported and five (7.94%) occurred in RA pregnancies. This is higher than that reported in a study by Chakravarty et al. in which IUGR in RA patients was 3.4% [20]. Rheumatic patients are also prone to anemia, with its occurrence linked to several factors, including inflammation-induced inhibition of erythropoiesis, imbalances in iron homeostasis, and treatment-related factors [21]. In the present study, there were three cases of anemia, two of which were related to SLE and one to RA. Moreover, patients with rheumatic disease face an increased risk of preeclampsia, although its exact cause remains elusive [22-23]. A nested case-control study within a cohort cited that the percentage of preeclampsia in pregnant patients with SLE ranged from 7.6% to 35% [24]. In our study, there were three (5.56%) SLE pregnancies with preeclampsia, which is less than the range previously suggested. A meta-analysis study revealed a significantly higher rate of venous thromboembolisms in inflammatory rheumatologic diseases, which was three times the rate of the general population [25]. However, In the current study, only one SS patient had a venous thromboembolism incidence.

In order to eliminate the predicted adverse events, the lowest possible dose (5 mg or less) of prednisolone was used to manage the disease in some cases. It was successful since prednisolone was safe and did not adversely affect birth weight. That was supported by Bandoli et al., which stated that adjusting the dose according to the disease activity and gestational age was not associated with low birth weight [26]. It has been found in previous studies that higher disease activity in SLE and RA is associated with low birth weight [27-28]. In this study, low birth weight was noticed in 43 infants, the vast majority were with mothers of SLE and RA. This observation was not attributed to disease activity or any other complication, which makes it difficult to conclude a result. The present study showed a high risk of preterm births, which was noticed in 25 pregnancies. Three, eight, and 14 cases were attributed to SS, RA, and SLE respectively. This finding is also in agreement with the study that showed there is an elevated risk for preterm birth in SLE, RA, systemic sclerosis (SSc), juvenile idiopathic arthritis (JIA), and polymyositis/dermatomyositis [29]. A study focused on RA patients revealed that 22.1% of preterm births occurred in the RA group while 8.4% occurred in the healthy control group [30]. In the present study, eight RA pregnancies (13.11%) resulted in preterm infants. This recorded value lies between the previously discussed affected group (22.1%) and the healthy group (8.4%). Moreover, preterm birth in our SS cases was 30%, which is relatively higher than the birth of preterm infants in both the controls (10%) and the SS cases (13%) found in a separate study [31]. Additionally, an investigation of patients by Al Arfaj and Khalil reported preterm births in 5.8% and 26.7% before and after SLE onset, respectively [32]. Our findings of preterm SLE births were similar to those of the affected group (26.42%). The same study [32] also revealed a 14.2% fetal loss rate before and a 29.7% fetal loss rate after the onset of SLE. In the current study, out of 54 SLE pregnancies, two (3.7%) ended in fetal loss which is lower than the previous ranges. 

A population's ideal rate of cesarian sections is between 10-15%, as indicated by the World Health Organization (WHO), and it is associated with a decrease in neonatal and maternal mortality. Moreover, WHO concluded that at the population level, cesarian section rates higher than 10% are not associated with reductions in maternal and newborn mortality rates [33]. In a previous study, the mode of delivery was assessed in patients with SS and healthy controls [31]. The results indicated that cesarian section deliveries were more common in SS cases (31%) compared to the control population (12%). In the current study, out of 11 SS pregnancies, six (54.55%) cases necessitated a cesarian section, which is relatively higher than the previous ranges. Our study also reported 20 SLE pregnancies that ended with a cesarian section as a mode of delivery rather than spontaneous vaginal delivery, which represents 37.04% of all SLE pregnancies that were reviewed. This finding and percentage are similar to the one observed in a study by Clowse et al. with an SLE percentage of 36.6% cesarian section, which could suggest that SLE is associated with a risk of cesarian section deliveries [34]. Furthermore, studies have reported a higher rate of pregnancy-related complications, specifically cesarian section in women with SLE compared to the general population and women with RA pregnancies [35], which is consistent with the findings of the present study as 19.05% of RA pregnant women delivered their babies with a cesarian section.

Literature showed that expectant females with rheumatological and autoimmune diseases were at more risk of experiencing both maternal and fetal complications [36]. However, only 16 babies in the current study (13.22%) required NICU admission, not contrary to the one reported by a study with regard to SS patients [37]. This indicates that with proper antenatal care and close monitoring by obstetricians and rheumatologists, a better outcome can be predicted. Moreover, our findings revealed that there is no difference between SLE with other comorbidities and RA or SS in terms of worse outcomes. However, it has been reported that SLE is associated with an increased risk of preterm labor, IUGR, pregnancy loss, and hypertensive diseases [38]. The differences between the findings could be explained by the close monitoring and high-quality care offered to our sample.

In our study, we observed postpartum complications, including bleeding and infection. The prevalence of postpartum infection in our cohort was 2.34%, less than the range reported in previous investigations, which suggested a prevalence of postpartum infections between 5% and 24% [39]. Similarly, the prevalence of postpartum bleeding in our study was 2.34%. This finding corresponds to other studies that commonly report postpartum bleeding rates of approximately 2-6% [40].

Some potential limitations are present in this study. First, this is a retrospective cohort study; therefore, we were unable to exclude selection and information bias. Second, the study was conducted in a tertiary single center that accepts more complicated cases rather than simple pregnancies. Thus, the women included in this study are not representative of all rheumatology patients at risk of developing pregnancy complications. Third, our study was conducted during the coronavirus disease 2019 (COVID-19) pandemic, and we believe there was difficulty in traveling between cities during the travel ban, making some patients receive obstetric care at local hospitals. Lastly, we used a small sample size, which may have limited the statistical power of our tests. To precisely analyze the outcome of each rheumatological group included in the present study, a larger sample size is needed. 

## Conclusions

Hormonal and physiological changes associated with pregnancy have an impact on chronic diseases. Controlling the disease before pregnancy, in addition to close follow-up during pregnancy, will result in a favorable outcome. A multidisciplinary team approach is crucial in such conditions and will deliver high-quality care. Educating the patient about the relationship between the disease and pregnancy is very integral. Lastly, reassuring the patient about the safety of medications during pregnancy will lead to improved medication adherence.
